# Psychological Antecedents of Healthcare Workers towards Monkeypox Vaccination in Nigeria

**DOI:** 10.3390/vaccines10122151

**Published:** 2022-12-15

**Authors:** Ramy Mohamed Ghazy, Debra Ukamaka Okeh, Malik Sallam, Mai Hussein, Horeya M. Ismail, Saja Yazbek, Amira Mahboob, Samar Abd ElHafeez

**Affiliations:** 1 Tropical Health Department, High Institute of Public Health, Alexandria University, Alexandria 21561, Egypt; 2 Epidemiology and Surveillance Unit, Caritas Nigeria, Abuja 900104, Nigeria; 3 Department of Pathology, Microbiology and Forensic Medicine, School of Medicine, The University of Jordan, Amman 11942, Jordan; 4 Department of Clinical Laboratories and Forensic Medicine, Jordan University Hospital, Amman 11942, Jordan; 5 Clinical Research Administration, Alexandria Directorate of Health Affairs, Egyptian Ministry of Health and Population, Alexandria 21554, Egypt; 6 Department of Biostatistics, High Institute of Public Health, Alexandria University, Alexandria 21561, Egypt; 7 Department of Biostatistics, Prime for Integrated Research Solutions, Alexandria 21561, Egypt; 8 Faculty of Public Health, Lebanese University, Beirut 6573, Lebanon; 9 Occupational Health and Industrial Medicine Department, High Institute of Public Health, Alexandria University, Alexandria 21561, Egypt; 10 Epidemiology Department, High Institute of Public Health, Alexandria University, Alexandria 21561, Egypt

**Keywords:** vaccination hesitancy, psychological 5C scale, vaccination policy, vaccine resistance, West Africa, monkeypox virus, outbreak

## Abstract

The ongoing monkeypox (MPX) outbreak has been declared a public health emergency of international concern. People in close contact with active MPX cases, including healthcare workers (HCWs), are at higher risk of virus acquisition since the MPX virus can be transmitted by skin contact or respiratory secretions. In this study, we aimed to assess the psychological antecedents of MPX vaccination among Nigerian HCWs using the 5C scale. We used an anonymous online cross-sectional survey to recruit potential participants using snowball sampling. The questionnaire aimed to assess the geo/socioeconomic features and the 5C psychological antecedents of vaccine acceptance (confidence, complacency, constraints, calculation, and collective responsibility). A total of 389 responses were included, with a median age of 37 years (IQR: 28–48), 55.5% males, and 60.7% married participants. Among the studied Nigerian HCWs, only 31.1% showed confidence in MPX vaccination, 58.4% expressed complacency towards vaccination, 63.8% perceived constraints towards MPX vaccination, 27.2% calculated the benefits and risks of vaccination, and 39.2% agreed to receive MPX vaccination to protect others. The determinants of MPX vaccine confidence were being single (OR = 5.07, 95% CI: 1.26–20.34, *p* = 0.022), a higher education level (with pre-college/high school as a reference, professional/technical: OR = 4.12, 95% CI: 1.57–10.73, *p* = 0.004, undergraduate: OR = 2.94, 95% CI: 1.32–6.55, *p* = 0.008, and postgraduate degree (OR = 3.48, 95% CI: 1.51–8.04, *p* = 0.003), and absence of chronic disease (OR = 2.57, 95% CI: 1.27–5.22, *p* = 0.009). The significant complacency predictors were having a middle-income (OR = 0.53, 95% CI: 0.33–0.89, *p* = 0.008), having a bachelor’s degree (OR = 2.37, 95% CI: 1.10–5.11, *p* = 0.027), and knowledge of someone who died due to MPX (OR = 0.20, 95% CI: 0.05–0.93, *p* = 0.040). Income was associated with perceived vaccination constraints (OR = 0.62, 95% CI: 0.39–0.99, *p* = 0.046). Participants aged 46–60 years had decreased odds in the calculation domain (OR = 0.52, 95% CI: 0.27–0.98, *p* = 0.044). Middle-income and bachelor degree/postgraduate education significantly influenced the collective responsibility domain (OR = 2.10, 95% CI: 1.19–3.69, *p* = 0.010; OR = 4.17, 95% CI: 1.85–9.38, *p* < 0.001; and OR = 3.45, 95% CI: 1.50–7.90, *p* = 0.003, respectively). An investigation of the 5C pattern-based psychological antecedents of MPX vaccination in a sample of Nigerian HCWs revealed low levels of vaccine confidence and collective responsibility with high levels of constraints and complacency. These psychological factors are recommended to be considered in any efforts aiming to promote MPX vaccination needed in a country where MPX is endemic.

## 1. Introduction

Monkeypox (MPX) is a re-emerging rare zoonotic infectious disease caused by a large double stranded DNA virus called the monkeypox virus (MPXV) [1]. MPXV belongs to the *Orthopoxvirus* genus and *Poxviridae* family, and it is more stable in detecting and repairing mutations than RNA viruses [2]. The MPXV was discovered in 1958 after two outbreaks in research-held monkeys began exhibiting symptoms of a pox-like illness [3]. The disease was first detected in humans in 1970, when a child in the Democratic Republic of the Congo was suspected of having smallpox. The first MPX outbreak outside of Africa was reported in the U.S. in 2003 [3,4].

The World Health Organization (WHO) Research and Development Blueprint designated MPX as an emerging illness in 2018 [5]. The growing global MPX outbreak was declared a public health emergency of international concern by the WHO Director-General on 23 July 2022 [6]. As of 23 November 2022, a total of 80,646 confirmed cases had been reported, with 53 deaths reported in 110 countries. Since 13 May 2022, a large proportion of these cases have been reported from countries where MPX transmission has not previously been documented [7]. This is the first time that cases and long-term transmission chains have been reported in countries with no direct or immediate epidemiological ties to West or Central Africa [8].

In Nigeria, MPX re-emerged in September 2017, with an outbreak reported in 14 states [9,10,11]. Overall, between September 2017 and October 2022, 2061 suspected cases have been reported from the 36 states and the Federal Capital Territory (FCT) [12,13]. Within the same period, 830 (40.3%) confirmed cases were reported from 32 states and the FCT. However, 1549 (75.5%) of the total suspected cases and 604 (72.8%) of the total confirmed cases in the country were reported between 1 January 2022–30 October 2022 [14,15]. Male preponderance was observed among the confirmed cases (550 male, 280 female), with a majority of the confirmed cases being reported in the largest city in the country; Lagos: 184 (22.2%), Rivers: 83 (10.0%), Bayelsa: 76 (9.2%), and Delta: 55 (6.6%) [16].

During the last week of October 2022 (24–30 October 2022), 90 new suspected MPX cases were reported from 16 Nigerian states and the FCT as follows: Plateau (20), Lagos (14), Bayelsa (9), Imo (8), Abia (7), FCT (5), Kaduna (5), Anambra (4), Ondo (4), Delta (3), Adamawa (2), Benue (2), Osun (2), Ogun (2), Edo (1), Niger (1), and Jigawa (1) [16,17]. Imported cases of MPX have recently been reported among U.S. travelers returning from Lagos and Ibadan, Nigeria [14]. Through an activated Emergency Operations Centre (EOC), the Nigeria Centre for Disease Control (NCDC) continues to coordinate the response. The NCDC has implemented measures to increase sample collection and testing [18]. Active public engagement is taking place to educate and raise awareness about preventive measures as well as allay public fear. Surveillance has been increased across the Federation, particularly in Bayelsa State [19].

Given the mild clinical symptoms associated with MPX, the US Food and Drug Administration (FDA) has approved the oral administration of two antiviral drugs, tecovirimat and brincidofovir, as well as intravenous injection of vaccinia immunoglobulin, for MPX treatment [1,20,21]. Nonetheless, vaccination has long been thought to be the most effective method of preventing and controlling infectious diseases such as MPX [1,22]. Previous research has shown that smallpox vaccination provides approximately 85% protection against MPXV infection [23,24].

Considering the rapid escalation in the number of MPX cases and its rapid spread globally, the Advisory Committee on Immunization Practices (ACIP) recommended MPX vaccination for most at-risk groups. These groups included health professionals responsible for taking care of MPX patients, including laboratory workers, research laboratory workers, and public health personnel responsible for the MPX outbreak response [1,25]. The ACIP recommended the use of JYNNEOS vaccine (a replication-deficient modified vaccinia Ankara vaccine), considering its efficacy and safety profiles compared to the earlier FDA-approved live vaccinia virus smallpox vaccine (ACAM2000) [26,27,28,29].

Given the likelihood that healthcare workers (HCWs) at all levels will be exposed to MPX in the course of their work, they should implement appropriate prevention and control measures during this MPX epidemic. In addition, HCWs and public health officials represent key groups that need to be prepared to take on their proper roles in disease surveillance, diagnosis, and management [30]. Vaccine hesitancy (VH) was named one of the top ten global health threats by the WHO in 2019. Psychological antecedents and beliefs of HCWs were significantly associated with their attitude towards coronavirus disease 2019 (COVID-19) and influenza vaccination [31,32,33]. The 5C scale is a validated tool that assesses the five psychological antecedents of vaccination and is effective in exploring VH among populations [34]. The main identified factors that affect the attitude of HCWs about vaccines included fear of the vaccine’s side effects, concerns related to safety, efficacy, and effectiveness, the short duration of the clinical trials, limited information, and social trust as the major drivers and reasons for their attitude toward vaccination [35].

To date, no study has investigated HCWs’ attitudes toward MPX vaccines in Nigeria. We think studying the psychological antecedents of HCWs may provide deeper insight about their attitude if the MPX vaccine becomes compulsory.

## 2. Materials and Methods

### 2.1. Study Design and Study Population

From 27 September 2022 to 4 November 2022, an anonymous online cross-sectional survey was conducted through social media platforms (Facebook, WhatsApp, and Twitter). The questionnaire was uploaded to Google forms, and the link was circulated among Nigerian HCWs. The study participants were recruited through the snowball sampling method. We included HCWs living in Nigeria, aged 18 years and above. The link was shared through social and work groups of HCWs in the country.

### 2.2. Sample Size

As there were no studies published in Nigeria to assess the attitude of HCWs towards the MPX vaccine, we assumed that 50.0% of the HCWs were willing to receive MPX vaccines. The sample size was calculated as follows: n = Z^2^ 1 − α/2P (1 − P)/e^2^ (n = the minimum number of respondents required; Z^2^ = (1.96)^2^ relative to the 95% confidence interval (CI); P = the estimated prevalence rate from the previous study; e = the required accuracy (5%)). The minimum sample size (n) for this study was 384 HCWs.

### 2.3. Data Collection Tool

The survey included the following sections: Section 1: sociodemographic characteristics (age, gender, nationality, living area, self-reported financial status (low vs. middle vs. high income), residence, level of education, marital status, occupation, and presence of comorbidities); with two yes/no questions asking if they were previously affected with MPX, if they have known anyone who died from MPX, and knowledge of various types of MPX vaccinations. Section 2: 15 questions covering the five 5C domains: confidence, complacency, constraints, calculation, and collective responsibility (1; strongly disagree to 5; strongly agree). To ensure single entries, a single response from each unique IP address was allowed. The survey’s first page included consent to participate, an explanation of the study’s research objectives, and assurances of confidentiality. It took 5–10 min to complete the questionnaire. The full questionnaire is provided in Supplementary File S1.

### 2.4. Operational Definitions

Suspected case: This is an acute illness with fever > 38.3 °C, intense headache, lymphadenopathy, back pain, myalgia, and intense asthenia, followed one to three days later by a progressively developing rash often beginning on the face (most dense) and then spreading elsewhere on the body, including the soles of the feet and the palms of the hands.

Confirmed case: This is any suspected case with laboratory confirmation—virus identification and detection by polymerase chain reaction (PCR), virus isolation [17].

Confidence: This term refers to trust in the vaccine, its dependability, and effectiveness [36], as well as trust in the health-care system and HCWs. Lack of trust and mistrust leads to lower vaccine uptake, decreased confidence in the health-care system, and increased acceptance of misinformation [37]. The confidence domain questions were: (1) I am completely confident that vaccines are safe; (2) vaccinations are effective; and (3) regarding vaccines, I am confident that public authorities will make decisions in the best interest of the community [38,39].

Constraint: This term refers to structural and psychological barriers that may prevent people from getting vaccinated even if they intend to do so. Access, time, self-efficacy, empowerment, and a lack of behavioral control are examples of such barriers [38]. Constraints domain questions included: (1) Everyday stress prevents me from getting vaccinated; (2) for me, it is inconvenient to receive vaccinations; (3) visiting the doctor’s office makes me feel uncomfortable; this keeps me from getting vaccinated [38,39].

Complacency: Responders have complacency if they perceive the risks of vaccine-preventable diseases as low and vaccination is not deemed a necessary preventive action. [40]. Complacency domain questions included: (1) Vaccination is unnecessary because vaccine-preventable diseases are not common anymore; (2) my immune system is so strong that it also protects me against diseases; and (3) vaccine-preventable diseases are not so severe that I should get vaccinated [38,39].

Calculation: It implies that people seek information in order to compare the risks of infections versus vaccination in order to make an informed decision. Calculation, it is argued, is a sign of risk aversion and may have a negative impact on vaccination behavior [38]. Calculation domain questions included: (1) When I think about getting vaccinated, I weigh benefits and risks to make the best decision possible; (2) for each and every vaccination, I closely consider whether it is useful for me; and (3) it is important for me to fully understand the topic of vaccination before I get vaccinated [38,39].

Collective responsibility: “the willingness to protect others through one’s own vaccination through herd immunity.” In other words, it refers to people who vaccinate themselves with the intention of protecting others and understand the role of herd immunity in limiting transmission [34]. Collective responsibility domain questions included: (1) When everyone is vaccinated, I do not have to get vaccinated too; (2) I get vaccinated because I can also protect people with a weaker immune system; and (3) vaccination is a collective action to prevent the spread of disease [38,39].

### 2.5. Statistical Analysis

SPSS version 21.0 was used for all statistical analyses. The number and percentage were used to express the demographic characteristics and questionnaire responses of the respondents. The relationship between independent variables (respondents’ demographic and sociological characteristics as well as their attitudes toward MPX vaccines) was evaluated using univariate analysis. The variables that received a *p* < 0.15 in univariate analysis were subjected to multivariate logistic regression analysis to determine the factors influencing their decision to receive MPX vaccines. The variables were described using odds ratios (OR) and 95% confidence intervals (CI), with *p* < 0.05 considered statistically significant.

The multivariate analysis estimated coefficients for each predictor included in the final model and adjusted them with respect to the other variables in the model to quantify the contribution of each predictor to the outcome. The overall model fit was evaluated by the likelihood ratio test and the omnibus test that showed an improvement over the null model (*p* < 0.05) in the five fitted models. The Cox and Snell R square and the Nagelkerke RsSquare provided an indication of the amount of variation in the dependent variable explained by each model. The statistical significance of individual regression coefficients (ßs) was tested using the Wald chi-square test (*p* < 0.050). The Hosmer–Lemeshow test explores whether the predicted probabilities are the same as the observed probabilities. An overall goodness of fit of the model is indicated by *p*-values > 0.050. The five fitted models produced a non-significant difference between the observed and predicted probabilities (*p* > 0.050) indicating a good model fit.

### 2.6. Ethics

The Ethics Committee of the High Institute of Public Health at Alexandria University approved this study (IRB No. 00012098/FWA No. 00018699). Furthermore, after outlining the study objective, research methods, and participants’ rights in the study, an online informed consent was obtained from all the participants before filling out the questionnaire.

## 3. Results

### 3.1. Participants’ Demographics

In this survey, we recruited 389 participants: 54.2% (*n* = 211) were males; 60.7% (*n* = 236) were married; 92% (*n* = 385) were living in urban areas; 63.5% (*n* = 247) had a low-income; 38.0% (*n* = 184) had a bachelor degree; 11.3% (*n* = 44) were complaining of chronic diseases; 8.2% (*n* = 32) had contracted MPX; and 4.1% (*n* = 16) knew someone who passed away due to MPX (Table 1).

### 3.2. Overall Results of 5C Psychological Antecedents for MPX Vaccination among Nigerian HCWs

**Figure 1:**
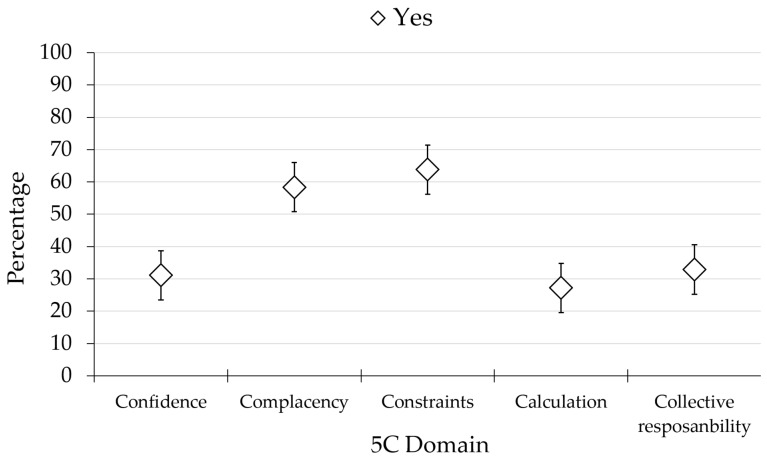
The 5C psychological antecedents of monkeypox vaccination in the study sample. Acceptance of each psychological domain is represented as an error bar.

Among the studied Nigerian HCWs, 31.11% showed confidence in MPX vaccination, 58.40% displayed complacency towards MPX vaccines, 63.80% perceived constraints in MPX vaccination, around one-fourth (27.2%) calculated the benefit and risk of vaccination, and 39.2% agreed to receive MPX vaccination to protect others (Figure 1).

#### 3.2.1. Confidence

Males were more confident regarding MPX vaccination compared to females (69.9% vs. 67.6%); however, this difference was not statistically significant (*p* = 0.630). Confidence was more common among married participants (74.8%) than others (*p* = 0.034). Residence was significantly associated with MPX vaccine confidence; people living in urban areas were more confident with vaccination than those living in rural areas (74.0% vs. 51.6%, *p* = 0.030). Participants with the highest educational level (postgraduate studies) and with the highest income had significantly higher vaccine confidence rates compared to others (75.2% and 85.7%, respectively). Having a history of chronic disease was inversely correlated with MPX vaccine confidence; about 47.7% of the participants with chronic disease were confident regarding MPX vaccination compared to 71.6% among those who did not report a history of chronic disease (*p* = 0.001). Previous infection with MPX and being aware of someone who got MPX infection were significantly associated with higher MPX vaccine confidence (87.5% for both, *p* < 0.050).

#### 3.2.2. Complacency

In the study sample, HCWs in the low-income level had the highest complacency levels (47.2%), compared to those of middle- and high-income levels (32.5% and 28.6%, respectively, *p* = 0.013). Compared to their peers, the HCWs who responded “I do not know” whether someone passed due to MPX infection had significantly higher levels of complacency toward MPX vaccination.

#### 3.2.3. Collective Responsibility

The HCWs in the middle-income group and those holding postgraduate degrees had significantly higher levels of collective responsibility than their colleagues (77.5% and 69.8%, respectively). The participants who did not report a history of chronic disease had higher levels of collective responsibility than those who had chronic diseases (52.3% vs. 69.0%, *p* = 0.026). Finally, participants who had contracted MPX infection but did not know someone who died due to MPX infection had higher levels of collective responsibility compared to others (84.4% and 73.3%, respectively, Table 2).

### 3.3. The Determinants of 5C Antecedents of MPX Vaccination in the Study Sample

The factors affecting the psychological antecedents of Nigerian HCWs about the MPX vaccine in five separate binary logistic regression models are illustrated in Table 3. Being a single participant was linked to five times higher odds of being more confident than other marital status categories (OR = 5.07, 95% CI: 1.26–20.34, *p* = 0.022). Additionally, having a higher education level and not having a chronic disease were significantly associated with increased odds of MPX vaccination confidence. The significant complacency predictors were having a middle-income (OR = 0.53, 95% CI: 0.33–0.89, *p* = 0.008), having a bachelor’s degree (OR = 2.37, 95% CI: 1.10–5.11, *p* = 0.027), and knowing someone who died due to MPX (OR = 0.20, 95% CI: 0.05–0.93, *p* = 0.040). Financial status was an important predictor related to vaccination constraints. Participants aged 46 to 60 years had decreased odds in the calculation domain (OR = 0.52, 95% CI: 0.27–0.98, *p* = 0.044). Middle-income and level of education (bachelor degree or postgraduate qualifications) significantly influenced the collective responsibility domain (OR = 2.10, 95% CI: 1.19–3.69, *p* = 0.010; OR = 4.17, 95% CI:1.85–9.38, *p* < 0.001; and OR = 3.45, 95% CI: 1.50–7.90, *p* = 0.003, respectively).

## 4. Discussion

The current 2022 MPX outbreak appeared to differ from the earlier outbreaks of the disease in endemic areas in terms of the groups considered high-risk for infection and the lower case-fatality rate; however, the burden of this emerging infection remains worrying [1,6,41,42]. The global risk of MPX is rated as moderate by the WHO [6]. Per region, the WHO rates the MPX risk in the Americas Region as high and moderate in the African Region, Eastern Mediterranean Region, European Region, and South-East Asia Region. The risk in the Western Pacific is rated as low [6]. On 20 October 2022, the International Health Regulation (IHR) Emergency Committee on the multi-country MPX outbreak held its third meeting. After taking into account the opinions of committee members and advisors as well as other factors in accordance with the IHR (2005), the WHO Director-General determined that this outbreak remains a public health emergency of international concern and issued revised temporary recommendations in relation to the outbreak [43]. Although the current outbreak is concentrated among men who have sex with men (MSM), HCWs are at risk of MPX acquisition due to their frontline role in taking care of the infected patients [44]. In addition, HCWs can be seen as a vulnerable group in outbreak settings with high prevalence of fear, anxiety, perceived risk and poor quality of life [45,46,47]. Therefore, the assessment of HCWs’ knowledge of MPX and their attitude toward its vaccination appears to be a timely and relevant issue [48,49,50,51,52,53].

Although the WHO does not deem mass MPX vaccination as a necessary step [54], vaccination prior to exposure is recommended for HCWs who are at high risk of MPX exposure [25]. It is well understood that increasing vaccination coverage is a critical tool for combating the ongoing spread of many infectious diseases, including MPX [55].

Although the current outbreak is concentrated among MSM [56], HCWs are at risk of MPX acquisition due to their frontline role in taking care of the infected patients [57]. Thus, the assessment of HCWs’ attitudes towards MPX vaccination is an important step in the efforts aiming to curb the rise in MPX cases [58]. Additionally, the attitude of HCWs towards vaccination can influence their vaccine recommendations to patients, besides the important role of HCWs in outbreak response and community education. Furthermore, the added value of MPX vaccination in Nigeria, which was considered an endemic country for MPX prior to the current 2022 outbreak, was demonstrated by 624 confirmed cases and 7 deaths due to the disease in 2022 alone [15].

Previous studies showed noticeable gaps in knowledge regarding the availability of MPX vaccination in different countries globally [50,51,52,59,60,61,62]. Nevertheless, the general attitude of HCWs towards MPX vaccination in endemic countries has not been pursued prior to the current study, to the best of our knowledge, despite the relevance of such an aim [48]. Before the ongoing 2022 MPX outbreak, the burden of the disease was substantial in endemic countries, including Nigeria, with reports of several outbreaks that were associated with mortalities [4,7].

As inferred through the 5C model, the attitude of the participants in the current study towards MPX vaccination can be considered unfavorable. Specifically, only 31% of the participants showed confidence in the MPX vaccination, while 58% displayed complacency in face of the dangers posed by the disease. Additionally, vaccine constraints were reported by 64% of HCWs in the study sample. Furthermore, only a third of the study sample showed a positive attitude towards MPX vaccination, in the context of collective responsibility. However, calculation as a determinant of attitude towards MPX vaccination involving weighing the benefits vs. risks of taking this preventive measure, was reported by a minority of participants (27%). The aforementioned results pointed to a generally negative attitude towards MPX vaccination among HCWs in Nigeria based on the 5C model for assessment of the psychological antecedents of vaccination. This can be translated into a majority of participants being reluctant towards MPX vaccination, as shown in four components of the model.

The current study findings contrasted the results of previous studies among HCWs in China, Italy, and Indonesia. Specifically, the recent study by Ricco et al. among physicians in Italy displayed a generally positive attitude towards the implementation of vaccination to prevent MPX, with 59% showing favorable attitudes toward this strategy [63]. An earlier study among Indonesian physicians showed a far more positive attitude towards the use of smallpox vaccination to prevent MPX, with 94% willing to get vaccinated [64]. Hong et al. found that 90% of Chinese HCWs were willing to get the MPX vaccine [65]. A less favorable attitude towards MPX vaccination was reported in a recent study that was conducted among clinicians in Ohio, U.S. [66]. In the study by Bates and Grijalva, less than half of the participants showed an intention to get the vaccine, and only 40% intended to deliver the MPX vaccination [66]. In line with our study findings, a recent study by Riad et al. among Czech HCWs showed that a majority of participants were either hesitant about MPX vaccination (46%), or rejected the vaccine (45%) [67]. Globally, a few studies investigated the attitude toward MPX vaccine acceptance, and a recent systematic review estimated the vaccine acceptance, particularly among physicians, at 64% [48]. This result appears much higher compared to our estimate, which may point to the importance of considering devising urgent plans to properly address this issue, including the need for more comprehensive studies in countries in the region.

In the current study, we aimed to decipher the psychological determinants of MPX vaccination. Our results pointed to the relevance of boosting both vaccine confidence and collective responsibility to enhance the positive attitude toward MPX vaccination. Confidence in vaccine effectiveness and safety have been reported as significant determinants of the willingness to receive various types of vaccines including COVID-19 and influenza vaccination [34,68]. The main identified predictors of confidence were higher education level, being single, and complaining of chronic diseases, while the chief determinants of collective responsibility were middle-income and higher educational level. On the other hand, in a large study that assessed confidence in the COVID-19 vaccine in 13 Arab countries, a higher education level was associated with reduced odds of vaccine confidence [34]. These findings would identify sub-populations that should be targeted with more focused intervention measures aiming to improve MPX vaccine acceptance. Therefore, the strategies aiming to promote MPX vaccination should consider the importance of educational campaigns highlighting the safety of these vaccines and their high effectiveness and value in disease prevention among this population. In addition, these campaigns should stress the importance of vaccination as a commitment to help in community protection, particularly for those vulnerable to severe disease (e.g., immunocompromised individuals). Such educational and vaccine promotion efforts are particularly needed in areas with high risk of outbreaks; therefore, the current study results can be used as an initial guide for the efforts aiming to reduce the risk of MPX in Nigeria [69].

Another important issue to be considered for the promotion of MPX vaccination is the challenge of vaccine constraints, including the requirement to pay for the vaccine and the availability of vaccination services that are comfortable for HCWs in terms of time and place. Indeed, high levels of constraints were found to be associated with a less favorable attitude towards MPX vaccination. We found that 63.8% displayed reluctance toward MPX vaccination due to constraints. The only significant determinant of reducing constraints as a barrier to MPX vaccination acceptance was a middle-income level versus a low-income level. This result highlights the significance of facilitating vaccination through measures including the free availability of vaccines and tailoring the time and place convenient for HCWs to get vaccinated [70]. Another important psychological determinant of MPX vaccination, as displayed by the results of the current study, was complacency. People who are complacent frequently believe that vaccination is unnecessary because their immune systems can protect them [39,71]. This result appeared understandable given the self-limited nature of MPX [1]. However, the disease burden in endemic countries, with a mortality rate previously reported at rates up to 10%, does not justify the high levels of complacency [7]. Thus, it is important to focus on educational efforts explaining the potential dangers of the disease and the benefits of implementing MPX vaccination for self-protection, as well as highlighting the role of collective responsibility among health professionals.

It is worth noting that having a chronic disease affected participants’ confidence and collective responsibility in bivariate analysis, but not in multivariate analysis. In this study, a large sector of Nigerian HCWs—58.4%—were complacent about the MPX vaccine. The main factors influencing such attitudes are having a middle-income and knowing someone who passed away due to MPX. This finding showed the importance of transparency and quality in reporting deaths related to MPX, as this can effectively improve the attitude of HCWs towards vaccination. Previous studies using the 5C model showed the significance of complacency as a predictor of vaccine acceptance and uptake among health professionals [31,32].

Finally, calculation as a psychological determinant of MPX vaccine acceptance was displayed by about one-fourth (27.2%) of the participants. The main determinant of such attitudes was the age group of the participants. The strategies focusing on calculation to improve vaccine acceptance among HCWs should focus on highlighting the safety of MPX vaccination among HCWs considered a high-risk group for contracting the disease, particularly for the newer generations of the vaccine, besides stressing the minimum risks associated with the vaccine uptake [72,73].

The current study results can be helpful as an initial guide if vaccine mandates become necessary as a strategy to contain the continuous emergence of MPX outbreaks. This is particularly relevant in West Africa, where low levels of COVID-19 vaccine acceptance were reported, which may point to a general attitude towards VH in the region [74,75]. Although the previous evidence showed that enforcing vaccination among HCWs can be a helpful strategy to contain infections, particularly for the benefit of patients who are at high risk of severe disease, this strategy may backfire if the necessary prerequisite steps of implementation are not properly considered and followed [76]. In addition, legal and ethical issues would arise if such measures were to be taken rather than considering other options, including providing financial incentives [77]. Thus, vaccine mandates can be spared for other approaches to be considered, including the focus on boosting trust in health authorities, and confidence in vaccine effectiveness and safety. In addition, the interventional measures should consider the importance of easy access to free vaccination and the role that HCWs can play in protecting vulnerable groups in society. Although the MPX risks appear low amid the ongoing outbreak, fatalities are being recorded as well as cases of severe disease, which should be considered among those who are complacent towards the disease.

### Strengths and Limitations

To the best of our knowledge, this is the first study to assess the psychological antecedents of Nigerian HCWs’ attitudes toward the MPX vaccine. Second, we used the validated 5C scale questionnaire, and this approach adds to the internal consistency and reliability of the study results. However, this study has several limitations, including the non-random sampling technique, that may affect the generalization of the study findings. Second, there are the inherent limitations of a cross-sectional survey itself. We are unable to address causality; the respondents are subject to reporting bias; and the results of this survey represent a single time point situation that may change overtime. Third, the subjective approach to the financial status of the participants was another caveat in the study. Fourth, the lack of details regarding the health professions and years of experience was a limitation that should be addressed in future studies, considering the importance of such variables in the decision to get vaccinated. Finally, the limited sample size should be taken into account as an additional shortcoming of the study. Nevertheless, the study can provide an initial guide and motivation for future studies that aim to better dissect the issue of MPX vaccination and its successful implementation, especially in the regions endemic to the virus.

## 5. Conclusions

Monkeypox, a disease endemic to Nigeria, has witnessed an increase in the number of cases with confirmed fatalities during the ongoing 2022 outbreak. However, Nigerian HCWs’ psychological antecedents for MPX vaccination pointed to an unsatisfactory attitude towards the vaccine. The participant HCWs in this study did not show the high levels of confidence and collective responsibility necessary for acceptance of the MPX vaccination. Moreover, high levels of constraints and complacency were reported in this study, which may be correlated with MPX vaccination hesitancy. Consequently, tailoring the efforts aiming to promote MPX vaccination among HCWs in Nigeria is needed for proper engagement in the vaccination program, hand in hand with targeting resistant groups, including those who are single, have low-income, and have low educational attainment. Targeting HCWs with proper intervention measures, including the provision of free vaccination and promoting confidence in vaccine effectiveness and safety, can consequently affect their vaccine recommendation as well, which can have an important implication for hindering community transmission of the virus.

## Figures and Tables

**Table 1:** Participants’ demographics and characteristics.

Characteristics	Frequency (%) (*N* = 389)
**Age**	
Median	37 (IQR) ^1^: 28–48
Range	18–68
**Gender**	
Male	211 (54.2)
Female	169 (43.4)
Prefer not to say	9 (2.3)
**Nationality**	
Nigerian	386 (99.1)
Dominican	1 (0.3)
Cuban	1 (0.3)
Spanish	1 (0.3)
**Marital status**	
Having a partner	25 (6.4)
Married	236 (60.7)
Single	115 (29.6)
Widow	13 (3.3)
**Living area**	
Urban	358 (92.0)
Rural	31 (8.0)
**Financial status**	
Low-income	247 (63.5)
Middle-income	118 (30.3)
High-income	19 (4.9)
Missing	5 (1.3)
**Education**	
Pre-college/High school	39 (10.0)
Professional/technical	53 (13.6)
Undergraduate (Bachelor)	148 (38.0)
Diploma	48 (12.3)
Postgraduate (Master)	74 (19.0)
Postgraduate (PhD)	27 (6.9)
**Chronic diseases**	
Yes	44 (11.3)
No	345 (88.7)
**Have you had monkeypox?**	
Yes	32 (8.2)
No	316 (81.2)
I do not know	41 (10.5)
**Has anyone that you know of died due to monkeypox?**	
Yes	16 (4.1)
No	232 (59.6)
I do not know	141 (36.2)

^1^ IQR: interquartile range; Each survey item is highlighted in bold.

**Table 2:** The 5C scale domains across different variables among the sample of Nigerian HCWs.

Variables	Confidence N (%)			Complacency N (%)			Constraints N (%)			Calculation N (%)			Collective Responsibility N (%)		
	Yes	No	OR (95% CI) *p*-Value	Yes	No	OR (95% CI) *p*-Value	Yes	No	OR (95% CI) *p*-Value	Yes	No	OR (95% CI) *p*-Value	Yes	No	OR (95% CI) *p*-Value
**Gender**			1.11 (0.72–1.71) 0.630			0.81 (0.54–1.21) 0.305			1.36 (0.89–2.06) 0.155			0.80 (0.51–1.26) 0.343			0.76 (0.49–1.1) 0.198
Male	151 (69.9)	65 (30.1)		85 (39.4)	131 (60.6)		85 (39.4)	131 (60.6)		153 (70.8)	63 (29.2)		139 (64.4)	77 (35.6)	
Female ^ref.^	117 (67.6)	56 (32.4)		77 (44.5)	96 (55.5)		56 (32.4)	117 (67.6)		130 (75.1)	43 (24.9)		122 (70.5)	51 (29.5)	
Age			0.215			0.617			0.561			0.085			0.008 *
18–30 ^ref.^	93 (73.2)	34 (26.8)		50 (39.4)	77 (60.6)		48 (37.8)	79 (62.2)		102 (80.3)	25 (19.7)		76 (59.8)	51 (40.2)	
31–45	101 (67.8)	48 (32.2)	0.77 (0.46–1.29)	66 (44.3)	83 (55.7)	1.23 (0.76–1.98)	55 (36.9)	94 (63.1)	0.96 (0.59–1.57)	105 (70.5)	44 (29.5)	0.59 (0.33–1.03)	113 (75.8)	36 (24.2)	2.11 (1.26–3.53)
46–60	60 (62.5)	36 (37.5)	0.61 (0.35–1.08)	41 (42.7)	55 (57.3)	1.15 (0.67–1.97)	30 (31.3)	66 (68.8)	0.75 (0.43–1.31)	63 (65.6)	33 (34.4)	0.47 (0.26–0.86)	58 (60.4)	38 (39.6)	1.02 (0.59–1.76)
>60	14 (82.4)	3 (17.6)	1.71 (0.46–6.31)	5 (29.4)	12 (70.6)	0.64 (0.21–1.93)	8 (47.1)	9 (52.9)	1.46 (0.53–4.05)	13 (76.5)	4 (23.5)	0.79 (0.24–2.65)	14 (82.4)	3 (17.6)	3.13 (0.86–11.45)
Marital Status			0.034 *			0.495			0.780			0.372			0.714
Having partner	15 (60.0)	10 (40.0)	2.40 (0.61–9.49)	9 (36.0)	16 (64.0)	1.88 (0.41–8.63)	9 (36.0)	16 (64.0)	0.90 (0.23–3.59)	17 (68.0)	8 (32.0)	1.33 (0.33–5.38)	15 (60.0)	10 (40.0)	0.94 (0.24–3.71)
Married	162 (68.6)	74 (31.4)	3.50 (1.11–11.07)	102 (43.2)	134 (56.8)	2.54 (0.68–9.46)	81 (34.3)	155 (65.7)	0.84 (0.27–2.64)	168 (71.2)	68 (28.8)	1.54 (0.49–4.89)	163 (69.1)	73 (30.9)	1.40 (0.44–4.41)
Single	86 (74.8)	29 (25.2)	4.75 (1.44–15.66)	48 (41.7)	67 (58.3)	2.39 (0.62–9.14)	46 (40.0)	69 (60.0)	1.07 (0.33–3.46)	90 (78.3)	25 (21.7)	2.25 (0.68–7.49)	75 (65.2)	40 (34.8)	1.17 (0.36–3.82)
Widow ^ref.^	5 (38.5)	8 (61.5)		3 (23.1)	10 (76.9)		5 (38.5)	8 (61.5)		8 (61.5)	5 (38.5)		8 (61.5)	5 (38.5)	
Living area			0.030 * 2.23 (1.06–4.67)			0.269 1.55 (0.71–3.39)			0.207 1.70 (0.74–3.91)			0.147 0.49 (0.18–1.31)			0.056 2.03 (0.97–4.26)
Urban	252 (70.4)	106 (29.6)		152 (42.5)	206 (57.5)		133 (37.2)	225 (62.8)		257 (71.8)	101 (28.2)		245 (68.4)	113 (31.6)	
Rural ^ref.^	16 (51.6)	15 (48.4)		10 (32.3)	21 (67.7)		8 (25.8)	23 (74.2)		26 (83.9)	5 (16.1)		16 (51.6)	15 (48.4)	
Financial Status			0.043 *			0.013 *			0.127			0.695			0.009 *
Low-income ^ref.^	161 (64.9)	87 (35.1)		117 (47.2)	131 (52.8)		99 (39.9)	149 (60.1)		179 (72.2)	69 (27.8)		153 (61.7)	95 (38.3)	
Middle-income	89 (74.2)	31 (25.8)	1.55 (0.96–2.52)	39 (32.5)	81 (67.5)	0.54 (0.34–0.85)	35 (29.2)	85 (70.8)	0.62 (0.39–0.99)	87 (72.5)	33 (27.5)	1.02 (0.62–1.66)	93 (77.5)	27 (22.5)	2.14 (1.30–3.52)
High-income	18 (85.7)	3 (14.3)	3.24 (0.93–11.31)	6 (28.6)	15 (71.4)	0.45 (0.17–1.19)	7 (33.3)	14 (66.7)	0.75 (0.29–1.93)	17 (81.0)	4 (19.0)	1.64 (0.53–5.04)	15 (71.4)	6 (28.6)	1.55 (0.58–4.14)
Education			0.001 *			0.177			0.578			0.175			0.001 *
Pre-college/High school ^ref.^	15 (38.5)	24 (61.5)		12 (30.8)	27 (69.2)		15 (38.5)	24 (61.5)		33 (84.6)	6 (15.4)		13 (33.3)	26 (66.7)	
Professional/technical	38 (71.7)	15 (28.3)	4.05 (1.68–9.77)	25 (47.2)	28 (52.8)	2.01 (0.84–4.79)	19 (35.8)	34 (64.2)	0.89 (0.38–2.10)	37 (69.8)	16 (30.2)	0.42 (0.15–1.20)	34 (64.2)	19 (35.8)	3.58 (1.50–8.55)
Bachelor	103 (69.6)	45 (30.4)	3.66 (1.76–7.63)	67 (45.3)	81 (54.7)	1.86 (0.88–3.95)	59 (39.9)	89 (60.1)	1.06 (0.51–2.19)	101 (68.2)	47 (31.8)	0.39 (0.15–0.99)	110 (74.3)	38 (25.7)	5.79 (2.71–12.39)
Post-graduate studies	112 (75.2)	37 (24.8)	4.84 (2.30–10.20)	58 (38.9)	91 (61.1)	1.43 (0.67–3.05)	48 (32.2)	101 (67.8)	0.76 (0.37–1.58)	37 (24.8)	112 (75.2)	0.55 (0.21–1.42)	104 (69.8)	45 (30.2)	4.62 (2.18–9.81)
Chronic diseases			0.001 * 2.76 (1.46–5.22)			0.280 1.44 (0.74–2.77)			0.516 1.25 (0.64–2.44)			0.716 1.14 (0.57–2.27)			0.026 * 2.03 (1.08–3.83)
Yes ^ref.^	21 (47.7)	23 (52.3)		15 (34.1)	29 (65.9)		14 (31.8)	30 (68.2)		31 (70.5)	13 (29.5)		23 (52.3)	21 (47.7)	
No	247 (71.6)	98 (28.4)		147 (42.6)	198 (57.4)		127 (36.8)	218 (63.2)		252 (73.0)	93 (27.0)		238 (69.0)	107 (31.0)	
Have you had MPX?			0.0183.41 (1.17–9.96)			0.105 0.52 (0.24–1.16)			0.539 0.78 (0.36–1.71)			0.123 2.13 (0.80–5.69)			0.030 * 2.84 (1.07–7.56)
Yes	28 (87.5)	4 (12.5)		9 (28.1)	23 (71.9)		10 (31.3)	22 (68.8)		27 (84.4)	5 (15.6)		27 (84.4)	5 (15.6)	
No/I do not know ^ref.^	240 (67.2)	117 (32.8)		153 (42.9)	204 (57.1)		131 (36.7)	226 (63.3)		256 (71.7)	101 (28.3)		234 (65.5)	123 (34.5)	
Knowing anyone who died due to MPX			0.165 3.28 (0.73–14.66)			0.016 * 0.19 (0.04–0.85)			0.137 0.39 (0.11–1.40)			0.837 1.13 (0.36–3.58)			0.346 0.62 (0.23–1.70)
Yes	14 (87.5)	2 (12.5)		2 (12.5)	14 (87.5)		3 (18.8)	13 (81.2)		12 (75.0)	4 (25.0)		9 (56.3)	7 (43.8)	
No/I do not know ^ref.^	254 (68.1)	119 (31.9)		160 (42.9)	213 (57.1)		138 (37.0)	235 (63.0)		271 (72.7)	102 (27.3)		252 (67.6)	121 (32.4)	

* denotes statistically significant values; MPX: monkeypox; N: number; ref.: reference category; OR: odds ratio; CI: confidence interval. *p*-values in bivariate analysis were calculated using the chi-squared test.

**Table 3:** Determinant of the 5C psychological antecedent of monkeypox vaccination among Nigerian healthcare workers in the study sample.

5C Domain, Variables	Category	OR	95% CI	*p* Value
**Confidence**				
Marital Status				0.025 *
	Widow	1		
	Having partner	1.8	(0.37–8.71)	0.465
	Married	2.73	(0.72–10.40)	0.142
	Single	5.07	(1.26–20.34)	0.022 *
Living area				
	Rural	1		
	Urban	1.63	(0.68–3.88)	0.274
Financial Status				0.107
	Low-income	1		
	Middle-income	1.42	(0.83–2.44)	0.205
	High-income	3.7	(0.96–14.07)	0.058
Education				0.015 *
	Pre-college/High school	1		
	Professional/technical	4.12	(1.57–10.73)	0.004 *
	Undergraduate	2.94	(1.32–6.55)	0.008 *
	Postgraduate degree	3.48	(1.51–8.04)	0.003 *
Chronic diseases	Yes	1		
	No	2.57	(1.27–5.22)	0.009 *
Have you had MPX?	No or do not know	1		
	Yes	2.3	(0.76–6.99)	0.141
Knowing anyone who died due to MPX				
	No or do not know	1		
	Yes	2.68	(0.57–12.67)	0.212
**Complacency**				
Income				0.017 *
	Low-income	1		
	Middle-income	0.53	(0.33–0.89)	0.008 *
	High-income	0.46	(0.16–1.25)	0.123
Education				0.161
	Pre-college/High school	1		
	Professional/technical	2.37	(0.99–5.72)	0.054
	Undergraduate	2.37	(1.10–5.11)	0.027 *
	Postgraduate degree	2.17	(0.99–4.79)	0.054
Have you had MPX?				
	No or do not know	1		0.271
		0.63	(0.27–1.44)	
	Yes			
Knowing anyone died due to MPX				
	No or do not know	1		
	Yes	0.2	(0.05–0.93)	0.040 *
**Constraints**				
Gender				
	Female	1		
	Male	1.36	(0.89–2.08)	0.156
Living Area				
	Rural	1		
	Urban	1.71	(0.74–3.98)	0.210
Financial Status				
	Low-income	1		0.132
	Middle-income	0.62	(0.39–0.99)	0.046 *
	High-income	0.77	(0.30–2.00)	0.593
Knowing anyone who died due to MPX				
	No or do not know	1		
	Yes	0.43	(0.12–1.56)	0.200
**Calculation**				0.251
Age				
	18–30	1		
	31–45	0.69	(0.386–1.24)	0.214
	46–60	0.52	(0.27–0.98)	0.044 *
	>60	0.75	(0.21–2.64)	0.654
Living area				
	Rural	1		
	Urban	0.59	(0.21–1.63)	0.309
Education				0.267
	Pre-college/High school	1		
	Professional/technical	0.49	(0.17–1.44)	0.195
	Undergraduate	0.48	(0.18–1.27)	0.138
	Postgraduate degree	0.72	(0.26–1.96)	0.520
Have you had MPX?				
	No or do not know	1		
	Yes	2.03	(0.75–5.51)	0.163
**Collective responsibility**				
Age				0.008 *
	18–30	1		
	31–45	1.74	(0.99– 3.06)	0.056
	46–60	0.67	(0.36–1.24)	0.201
	>60	3.19	(0.55–18.45)	0.195
Living area				
	Rural	1		
	Urban	1.41	(0.6–3.32)	0.431
Financial Status				0.031 *
	Low-income	1		
	Middle-income	2.1	(1.19–3.69)	0.010 *
	High-income	0.91	(0.22–3.72)	0.890
Education				0.006 *
	Pre-college/High school	1		
	Professional/technical	2.44	(0.97–6.17)	0.057
	Undergraduate	4.17	(1.85–9.38)	0.001 *
	Postgraduate degree	3.45	(1.50–7.90)	0.003 *
Chronic diseases				
	Yes	1		
	No	1.82	(0.899–3.673)	0.096
Have you had MPX?				
	No or do not know	1		
	Yes	2.01	(0.69–5.82)	0.198
Knowing anyone who died due to MPX				
	No or do not know	1		
	Yes	0.42	(0.14–1.29)	0.118

* denotes statistically significant values; MPX: monkeypox; CI: confidence interval. Each 5 C item is highlighted in bold.

## Data Availability

All data supporting the results of this study are available upon reasonable request from the first corresponding author (R.M.G.).

## Supplementary Materials

The following are available online at https://www.mdpi.com/article/10.3390/vaccines10122151/s1, Supplementary File S1: The study questionnaire was translated into English.
