# Supplementary material for: Psychological Antecedents of Healthcare Workers towards Monkeypox Vaccination in Nigeria
Source: Vaccines (Basel). 2022 Dec 15;10(12):2151. doi: 10.3390/vaccines10122151 (PMC9783513; doi:10.3390/vaccines10122151)
Supplement: Supplementary file 1 [file vaccines-10-02151-s001.zip › vaccines-2092201-supplementary.pdf]

Psychological Antecedents of Healthcare Workers Towards Monkeypox Vaccination in Nigeria

Supplementary File S1

You are invited to participate in a paper entitled "Evaluation of Psychological Drivers of Monkey Pox Virus Vaccines in Nigeria". This research aims to assess the public's beliefs regarding the monkeypox vaccine. This research is aimed at the general public and healthcare workers over the age of 18 years. The results of the current research may provide an opportunity to develop an awareness plan about the monkeypox vaccine in order to enhance knowledge and dispel myths about it. Your participation in this survey is voluntary and you are free to withdraw from this study at any time you wish. This study has been approved by the College of Medicine Ethics Committee No. 00018699 and all data will be anonymized without any indication of your personal identity.

Monkeypox, a disease that is spreading in the world We, as doctors and researchers from several universities, hospitals and medical centers, invite you to participate in this study, which aims to assess the psychological motives for vaccinations against monkeypox.

Agreement status

Participate in this study:* Required item

- Agree
- Disagree

Socio-demographics questions:

- Gender
  - Male
  - Female
  - Prefer not to say
- Age in years …….
- Nationality …….
- Country where you are living now: …….
- Marital status:
  - Single
  - Married
  - Having a partner
  - Widow
- Add living area:
  - Urban
  - Rural
  - Other: ……
- Financial status (self-assessment)
  - Low income
  - Middle income
  - Upper income
- What is the highest level of education/degree you have completed?
  - Pre-college/High school
  - professional/technical
  - Undergraduate (Bachelor)
  - Diploma
  - Postgraduate (Master)
  - Postgraduate (PhD)
- Do you suffer from any chronic diseases?
  - Yes
  - No
- Occupation
  - Health Care Worker (HCW)
  - Working
  - Not-working
  - Student in health or medical faculty
  - Student in other faculties

- Have you had Monkeypox?
  - Yes
  - No
  - I do not know
- Has anyone died due to Monkeypox, that you know of?
  - Yes
  - No
  - I do not know

Please answer these questions about Monkeypox vaccinations:

- I am completely confident that vaccines are safe
  - strongly agree
  - agree
  - neutral
  - disagree
  - strongly disagree
- Vaccinations are effective
  - strongly agree
  - agree
  - neutral
  - disagree
  - strongly disagree
- Regarding vaccines, I am confident that public authorities decide in the best interest of the community
  - strongly agree
  - agree
  - neutral
  - disagree
  - strongly disagree
- Vaccination is unnecessary because vaccine preventable diseases are not common any more
  - strongly agree
  - agree
  - neutral
  - disagree
  - strongly disagree
- My immune system is so strong, it also protects me against diseases
  - strongly agree

- agree
- neutral
- disagree
- strongly disagree

- Vaccine-preventable diseases are not so severe that I should get vaccinated
  - strongly agree
  - agree
  - neutral
  - disagree
  - strongly disagree
- Everyday stress prevents me from getting vaccinated
  - strongly agree
  - agree
  - neutral
  - disagree
  - strongly disagree
- For me, it is inconvenient to receive vaccinations
  - strongly agree
  - agree
  - neutral
  - disagree
  - strongly disagree
- Visiting the doctors' makes me feel uncomfortable; this keeps me from getting vaccinated
  - strongly agree
  - agree
  - neutral
  - disagree
  - strongly disagree
- When I think about getting vaccinated, I weigh benefits and risks to make the best decision possible
  - strongly agree
  - agree
  - neutral
  - disagree
  - strongly disagree
- For each and every vaccination, I closely consider whether it is useful for me
  - strongly agree

- agree
- neutral
- disagree
- strongly disagree

- It is important for me to fully understand the topic of vaccination, before I get vaccinated
  - strongly agree
  - agree
  - neutral
  - disagree
  - strongly disagree
- When everyone is vaccinated, I don't have to get vaccinated too
  - strongly agree
  - agree
  - neutral
  - disagree
  - strongly disagree
- I get vaccinated because I can also protect people with a weaker immune system
  - strongly agree
  - agree
  - neutral
  - disagree
  - strongly disagree
- Vaccination is a collective action to prevent the spread of disease
  - strongly agree
  - agree
  - neutral
  - disagree
  - strongly disagree

Do you have any idea about the various types of Monkeypox vaccinations?

- Yes
- No
